# Atezolizumab‐related sclerosing cholangitis with multiple liver abscesses in a patient with lung squamous cell carcinoma: A case report

**DOI:** 10.1002/rcr2.1324

**Published:** 2024-03-13

**Authors:** Daisuke Jingu, Akira Horii, Takehiro Yajima, Ryuta Ohira, Satoshi Ubukata, Kosuke Satou, Hiroshi Takahashi, Hiroshi Watanabe, Hiroyuki Funayama

**Affiliations:** ^1^ Department of Respiratory Medicine Saka General Hospital Shiogama Japan; ^2^ Department of Internal Medicine Saka General Hospital Shiogama Japan; ^3^ Department of Gastroenterology and Hepatology Saka General Hospital Shiogama Japan

**Keywords:** atezolizumab, immune checkpoint inhibitor, immune‐related adverse events, liver abscess, sclerosing cholangitis

## Abstract

A 76‐year‐old man underwent an operation for lung squamous cell carcinoma in the right lower lobe, followed by initial adjuvant therapy with atezolizumab, an antibody against anti‐programmed death‐ligand 1 (PD‐L1). On day 4 after atezolizumab treatment, the patient developed general malaise and fatigue. He was diagnosed with atezolizumab‐induced sclerosing cholangitis. Steroid treatment was started, and patient's condition, including symptoms, laboratory data and imaging findings, improved. Antibiotic treatments were ended on day 40, and the steroid dose was gradually reduced. Multiple liver abscesses were observed on day 106, and another treatment with antibiotics became necessary. The patient eventually recovered from liver abscesses. Sclerosing cholangitis induced by immune checkpoint inhibitor is rare, and the long‐term clinical data about this adverse effect is limited. Hence, we think it is important to raise an alarm over sclerosing cholangitis coupled with liver abscesses after immunosuppressive therapy.

## Graphical abstract

This report highlights the long‐term clinical data from a case of immune checkpoint inhibitor (ICI) related sclerosing cholangitis (SC) with multiple liver abscesses. ICI‐associated SC is rare, and pathological condition and/or long‐term follow‐up data are limited. Hence it is very important to share our experience with many medical professions in order to understand and investigate irAEs and find better ways for clinical management of patients.

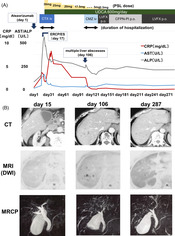

## INTRODUCTION

Recent development of immune checkpoint inhibitors (ICIs) has impacted the treatment of lung cancer. Atezolizumab, an antibody against anti‐programmed death‐ligand 1 (PD‐L1), is an ICIs that shows significant benefits for patients with metastatic non‐small‐cell lung cancer (NSCLC) without treatable driver mutations. In addition, treatment with atezolizumab after adjuvant chemotherapy can result in disease‐free survival in patients with resected NSCLC.^1^

With the widespread use of ICIs, immune‐related adverse events (irAEs) such as thyroid dysfunction, inflammatory dermatitis, colitis, and pneumonitis have been reported.^1^ We experienced a long‐term follow‐up case with lung cancer who developed sclerosing cholangitis (SC) coupled with multiple liver abscesses after treatment with atezolizumab. Because ICI‐induced SC is extremely rare, and its detailed clinical course remains unclear, this case is worth reporting to the public.

## CASE REPORT

A 76‐year‐old man who suffered from lung squamous cell carcinoma in the right lower lobe underwent a right lower lobectomy. He had concomitant diseases with asthma and chronic obstructive pulmonary disease, but he had no past medical history of the auto immune disease, no other possible risk factors associated with other biliary diseases including sclerosing cholangitis. Final diagnosis after the operation was lung squamous cell carcinoma Stage IIIA (pT2bN2M0). Tumour proportion score was over 50% in the resected specimen. Mutation analyses with Oncomine Dx Target Test multi CDx system (Thermo Fisher Scientific Inc., Waltham, MA) were performed, but no draggable mutations were observed.

**FIGURE 1:**
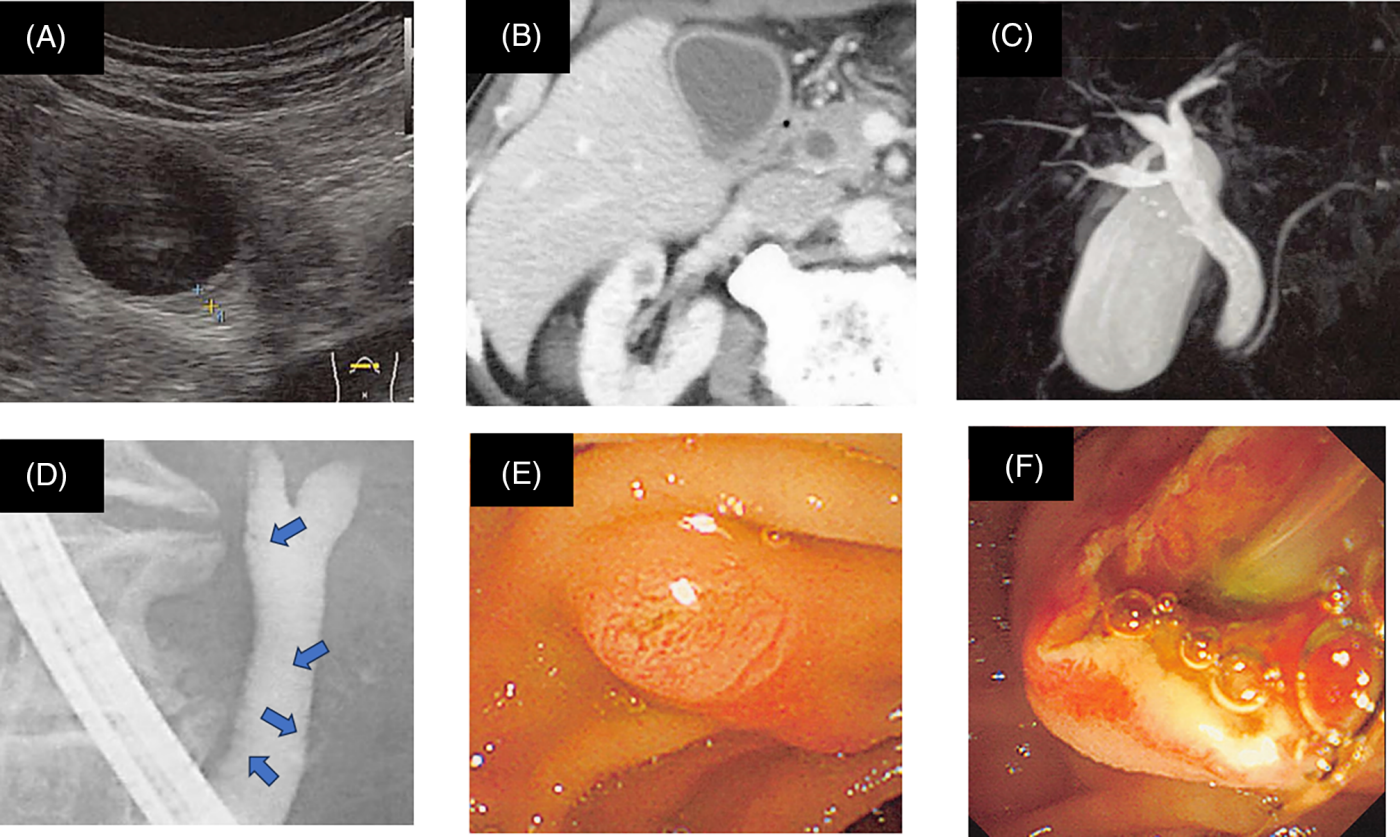
Imaging and endoscopic findings from the patient demonstrated immune‐related sclerosing cholangitis and acute obstructive suppurative cholangitis. (A) Ultrasound sonography and (B) computed tomography showed dilation of the common bile duct and thickening of gallbladder wall without stones. (C) Magnetic resonance cholangiopancreatography revealed dilation of the common bile duct and irregularly narrowed intrahepatic bile ducts. (D) Endoscopic retrograde cholangiopancreatography showed biliary sludges (indicated by arrows). (E) Endoscopic finding showed protrusion of papilla of Vater. (F) After sphincterotomy, outflow of minute biliary sands with purulent fluid was observed.

As adjuvant chemotherapy, the patient first received 4 courses of cisplatin plus vinorelbine, followed by the treatment with atezolizumab. On day 4 (after administration of atezolizumab), the patient developed general malaise and fatigue. By day 15, patient's symptoms persisted, and serum levels of liver enzymes were slightly elevated (Table 1). Computed tomography (CT), ultrasound sonography, and magnetic resonance cholangiopancreatography (MRCP) showed dilation of the common bile duct and thickened gallbladder wall without stones in gallbladder and/or bile duct (Figure 1A–C). Either obstructive cholangitis due to papillary obstruction with/without minute bile duct stones or irAEs induced by atezolizumab were suspected. Antibiotic therapy was initiated, but fever persisted and laboratory data worsened. Endoscopic retrograde cholangiopancreatography performed on day 17 demonstrated biliary sludges (Figure 1D) that suggested obstruction at the papilla of Vater with minute biliary sludges. We then performed an endoscopic sphincterotomy (ES) and observed the outflow of minute biliary sands with purulent fluid (Figure 1E,F).

**TABLE 1:** Relevant laboratory data on days 15 and 106 after administration of atezolizumab.

Biochemistry	Day 15	Day 106	Haematology	Day 15	Day 106	Serology	Day 15
CRP	2.55	2.66 mg/dL	WBC	4600	8000 /μL	HCV Ab	(−)
AST	45	35 U/L	Neut	56.6	78.0%	HBs Ag	(−)
ALT	41	75 U/L	Lymph	17.0	14.1%	HBs Ab	(−)
ALP	182	341 U/L	Mono	12.6	6.8%	HBe Ab	(−)
γ‐GTP	113	316 U/L	Eo	13.5	0.5%	CMV‐IgM	0.26 S/CO
T‐Bil	0.5	0.7 mg/dL	Hb	9.9	11.5 g/dL	CMV‐IgG	65.2 AU/mL
LDH	181	182 U/L	Plt	21.2	23.6 ×10^4^/μL	EBV VCA IgM	< x10
CK	31	15 U/L	Coagulation			EBV VCA IgG	×80
Na	138	140 mEq/L	PT‐%	81.2	110.4%	EBV EBNA	×10
K	4.3	4.1 mEq/L	PT‐INR	1.11	0.96	Anti‐LKM‐1 Ab	<5.0
Cl	103	99 mEq/L	APTT	28.8	24.5 s	IgG	1201 mg/dL
Ca	8.6	9.3 mg/dL	D‐dimer	2.3	0.5 μg/mL	IgG4	21.8 mg/dL
BUN	29.1	23.4 mg/dL	Blood Sugar			ANA	< x40
Cre	0.91	0.92 mg/dL	BS	129	151 mg/dL	Anti‐mit Ab	< x20
AMY	52	71 U/L	A1c	5.5	N.D. %	Anti‐mit. M2 Ab	2.1
Alb	3.3	3.2 g/dL				MPO‐ANCA	(−)
TP	6.1	N.D. g/dL				PR3‐ANCA	(−)
eGFR	62.10	61.32 mL/min/1.73m^2^					

Abbreviation: ND, denotes not determined.

**FIGURE 2:**
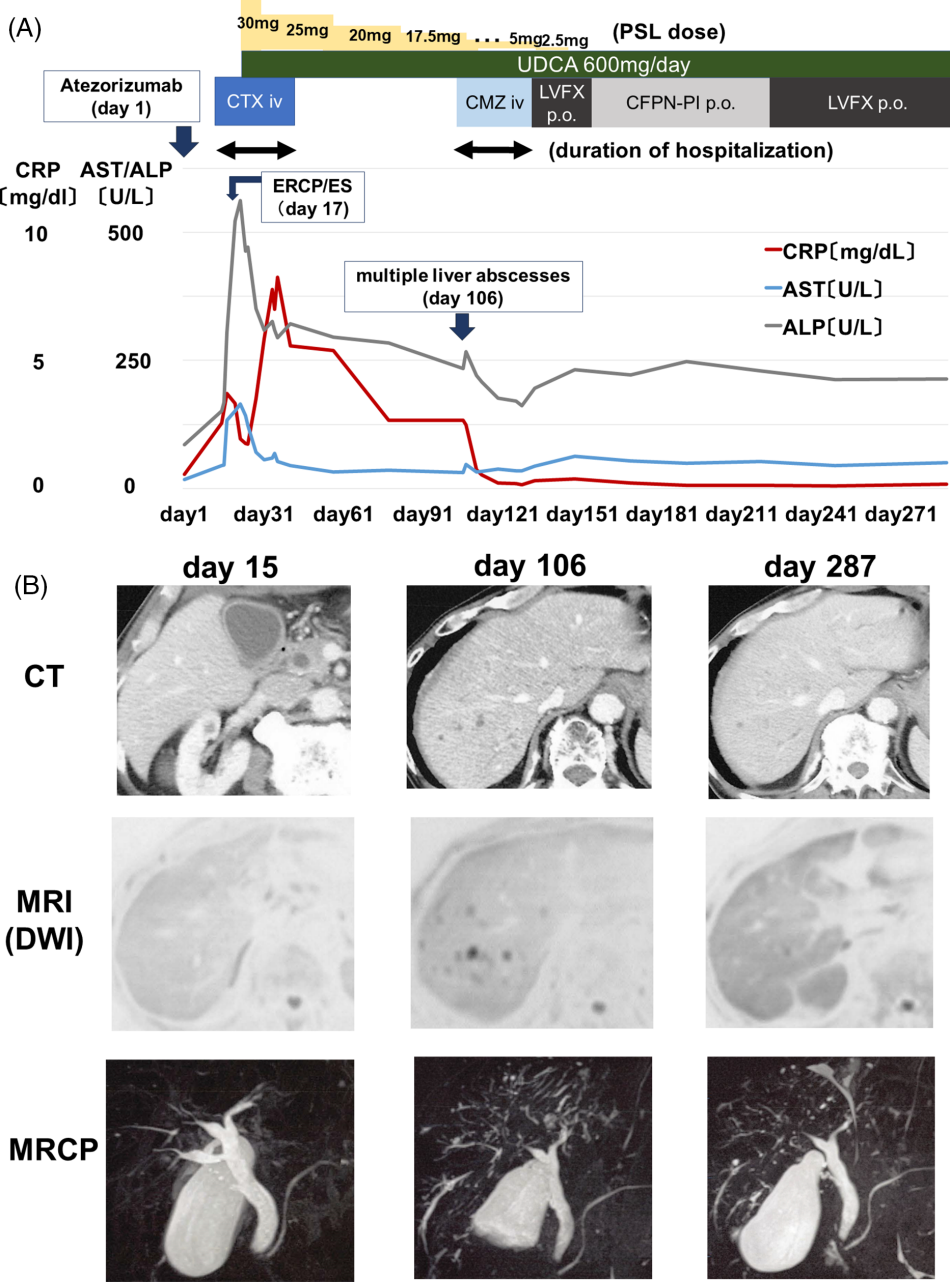
Clinical course with treatments and imaging data are shown. (A) Agents for treatment, doses, and timeline chart of CRP, AST, and ALP are shown. Relevant events are also indicated. PSL was tapered from 17.5 mg/day by 2.5 mg every 3 days, and from 5.0 mg/day by 2.5 mg every 3 weeks. Stepwise reduction of PSL dose from 17.5 mg/day to 5 mg/day is indicated by “…”. Double‐headed arrows indicate duration of hospitalization. (B) Images of CT, MRI (DWI), and MRCP on days 15, 106, and 287 are shown; day 15 was the onset of the irAE‐SC, day 106 was the onset of multiple liver abscesses, and day 287 was after improvement of liver abscesses. ALP, alkaline phosphatase; AST, aspartate aminotransferase; CFPN‐PI, cefcapene pivoxil; CMZ, cefmetazole sodium; CRP, C‐reactive protein; CT, computed tomography; CTX, cefotaxime; DWI, diffusion‐weighted imaging; ERCP, endoscopic retrograde cholangiopancreatography; ES, endoscopic sphincterotomy; LVFX, levofloxacin; MRCP, magnetic resonance cholangiopancreatography; MRI, magnetic resonance imaging; PSL, prednisolone; UDCA, ursodeoxycholic acid.

After ES, neither the fever nor laboratory data improved (Figure 2A). Atezolizumab‐induced sclerosing cholangitis was strongly suspected. Steroid treatment was started at a dose of 30 mg/day (0.5 mg/kg/day) of prednisolone (PSL). The patient's condition, including symptoms, laboratory data, and imaging findings gradually improved. We halted antibiotic treatment and started steroid tapering. The patient's symptoms did not recur, and he was discharged on day 32, 21 days after administration of steroids.

C‐reactive protein (CRP) and hepatobiliary enzyme levels were increased again at 2 months after the initiation of steroid therapy when PSL was 17.5 mg/day (see Table 1 and Figure 2A) without notable clinical symptoms including fever onset. A contrast‐enhanced CT scan and MRI showed multiple non‐enhanced nodules in the liver, and MRCP indicated tension in the common bile duct and contraction of the gallbladder on day 106 (Figure 2B). In addition, partial stricture and dilation of the intraductal bile ducts had worsened. We assumed that the combination of partial stricture by irAE‐SC, immunosuppression by steroid, and retrograde biliary infections by ES induced multiple liver abscesses. PSL tapering was done very carefully; from 17.5 mg/day by 2.5 mg every 3 days, and from 5.0 mg/day by 2.5 mg every 3 weeks. Treatments with other antibiotics and the taper‐off steroid were effective, and the patient recovered from liver abscesses (Figure 2B).

Neither the patient's irAE‐SC nor his liver abscesses recurred at 6 months after finishing antibiotics, and no signs of lung cancer recurrence were observed.

## DISCUSSION

Atezolizumab is one of the key drugs for NSCLC in the advanced stages and adjuvant therapy.^1^ IrAE‐SC is reported mainly from patients treated by anti‐programmed cell death‐1 (PD‐1) such as nivolumab^2^ or pembrolizumab,^3^, ^4^ but atezolizumab is not an exception.^5^ The incidence of SC related to atezolizumab is estimated as 0.046% (1 of 2140 treated patients with NSCLC) according to post‐marketing surveillance from April 2018 to October 2018 by the Ministry of Health, Labour and Welfare in Japan (https://chugai‐pharm.jp/content/dam/chugai/product/tec/div/survey/doc/tec_report1901.pdf, in Japanese).

Sato et al. proposed the possibility of pembrolizumab associated SC with cytotoxic T cells (CTLs) that are clusters of differentiation (CD) 8‐positives. Prominent infiltration of CTLs at the portal area were evident at the onset of the pembrolizumab‐induced irAE‐SC, but CTL reduction was observed after recovery of irAE‐SC.^4^

In general, development of irAE‐SC occurs rather late, mostly after the 5th course of ICI or even after treatment termination.^5^ Our case, however, developed irAE‐SC just after the first administration of atezolizumab. Thus, some patients may develop irAE‐SC precociously, and we should monitor them very carefully for a long period from immediately after ICI administration.

It is not yet possible to predict individuals who are at risk of developing irAE‐SC. The immune response may play a role. If this is the case, then a genetic background such as HLA may be associated with irAE‐SC.

ICI‐associated SC is rare, and pathological condition and/or long‐term follow‐up data are limited. Only one case report of pembrolizumab‐related SC with multiple liver abscesses during treatment is available presently.^3^ ICIs are rather new: we need to accumulate our knowledge of irAE‐SC. Hence it is very important to share our experience with many medical professionals to investigate irAEs and find better ways to clinically manage patients.

## AUTHOR CONTRIBUTIONS

DJ, TY, RO, SU, KS, HT, HW, and HF substantially contributed to the diagnosis and clinical care of the patient. DJ wrote the draft of the manuscript, and DJ and AH critically revised it for important intellectual content.

## CONFLICT OF INTEREST STATEMENT

None declared.

## ETHICS STATEMENT

The authors declare that appropriate written informed consent was obtained for the publication of this manuscript and accompanying images.

## Data Availability

The data that support the findings of this study are available from the corresponding author upon reasonable request.
